# Platinum(II) and Palladium(II) Complexes of Pyridine-2-Carbaldehyde Thiosemicarbazone as Alternative Antiherpes Simplex Virus Agents

**DOI:** 10.1155/2007/56165

**Published:** 2007-02-14

**Authors:** D. Kovala-Demertzi, T. Varadinova, P. Genova, P. Souza, M. A. Demertzis

**Affiliations:** ^1^Sector of Inorganic and Analytical Chemistry, Department of Chemistry, University of Ioannina, 45110 Ioannina, Greece; ^2^Laboratory of Virology, Faculty of Biology, Sofia University “St. Kliment Ohridski”, 8 Dragan Tzankov Boulevard, 1164 Sofia, Bulgaria; ^3^Laboratory of Cell Cultures, Department of Virology, National Centre of Infectious and Parasitic Diseases, 44A Stoletov Boulevard, 1233 Sofia, Bulgaria; ^4^Departamento de Química Inorgánica, Facultad de Ciencias, Universidad Autónoma de Madrid, C/ Francisco Tomás y Valiente 7, 28049 Madrid, Spain

## Abstract

The cytotoxicity and the antivirus activity of Pd(II) and Pt(II) complexes with pyridine-2-carbaldehyde thiosemicarbazone (HFoTsc) against HSV replication were evaluated on four HSV strains—two *wt* strains Victoria (HSV-1) and BJA (HSV-2) and two ACV^R^ mutants with different *tk* gene mutations R-100 (TK^A^, HSV-1) and PU (TK^N^, HSV-2). The experiments were performed on continuous MDBK cells and four HSV 1 and HSV 2 strains were used, two sensitive to acyclovir and two resistant mutants. The five complexes of HFoTsc, [Pt(FoTsc)Cl], [Pt(FoTsc)(H_2_FoTsc)]Cl_2_, [Pt(FoTsc)_2_], [Pd(FoTsc)(H_2_FoTsc)]Cl_2_, and [Pd(FoTsc)_2_], were found to be effective inhibitors of HSV replication. The most promising, active, and selective anti-HSV agent was found to be complex [Pt(FoTsc)(H_2_FoTsc)]Cl_2_. This complex could be useful in the treatment of HSV infections, since it is resistant to ACV mutants. PCR study of immediate early 300 bp ReIV Us1 region reveals that the complex [Pt(FoTsc)(H_2_FoTsc)]Cl_2_ specifically suppressed *wt* HSV-1 genome 2 hours after the infection, not inducing apoptosis/necrosis on the 8 hours after virus infection. The target was found to be most probably the viral, instead of the host cell DNA.

## 1. INTRODUCTION

Herpes simplex viruses (HSVs) are highly adapted human pathogens with rapid lytic cycle and ability to invade sensory neurons. The primary agents of recurrent facial and genital herpes lesions are HSV-1 and HSV-2 while genital herpes (GH) is the most common sexually transmitted infection in the world [1–3]. Moreover, GH is the main factor of increasing three to five times the risk of HIV transmission, stimulating HIV replication, and finally leading to the progression of AIDS [4–6]. Acyclovir (ACV) is a prodrug and it is the first nucleoside-based therapeutic effective for the treatment of primary and recurrent HSV infections [7]. Effective HSV suppression with ACV indirectly lowers the HIV load. ACV has to be phosphorylated by the viral thymidine kinase (TK) and subsequently by cellular kinases in order to inhibit competitively HSV DNA polymerase and to terminate the viral DNA chain elongation. However, under systematic administration, resistant mutants appeared with high frequency and their main sources are immune-compromised individuals [7–9]. The two most common causes of resistance are mutations in thymidine kinase (TK) gene, approximately 95% to 96% of ACV-resistant (ACV^R^) HSV isolated are thymidine-kinase-(TK-)deficient (TK^N^) or TK-partial (TK^P^) and the remaining isolates are usually TK-altered (TK^A^) mutants unable to phosphorylate the prodrug but not the thymidine [9]. The problem for effective treatment of HSV infections is still open, since the resistance to ACV and the cross-resistance to other nucleoside analogs increase with relatively high frequency.

The earliest antivirals are thiosemicarbazones, Tscs. The bioactivity of Tscs is due to the inhibition of ribonucleotide reductase (RR) and due to complexation with essential metals [10–12]. The activity of Pt(II) and Pd(II) complexes of pyridine-2-carbaldehyde (HFoTsc) against the replication of wild type (*wt*) HSV-1, was recently referred to by Varadinova et al. [13]. The antiviral activity of platinum complexes with antiviral agents acyclovir, penciclovir, and famciclovir has been recently reported [14–16].

The aim of the present study was to evaluate comparatively the activity of complexes of Pd(II) and Pt(II) with pyridine-2-carbaldehyde thiosemicarbazone (HFoTsc) against HSV replication. Special attention was given to the efficacy of compounds against ACV^R^ viruses.

## 2. EXPERIMENTAL

### 2.1. Metal complexes

**Scheme 1:**
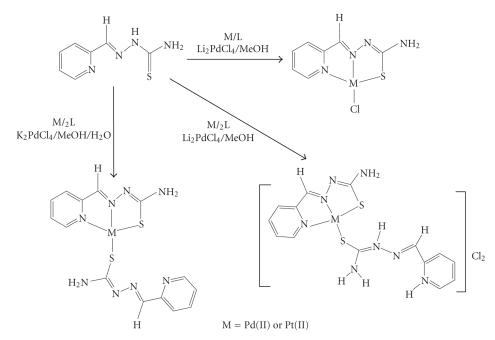
The reaction scheme for the synthesis of the complexes of Pd(II) and Pt(II) with FoTsc {[M(FoTsc)Cl], [M(FoTsc)(H_2 _FoTsc)]Cl_2_, [M(FoTsc)_2_]} [19, 20].

Solvents were purified and dried according to standard procedures. The ligand HFoTsc, **1**, and the complexes of Pt(II) and Pd(II) [PtCl(FoTsc)], **2**, [Pt(FoTsc)(H_2_FoTsc)]Cl_2_, **3**, [Pt(FoTsc)_2_], **4**, [PdCl(FoTsc)], **5**, [Pd(FoTsc)(H_2_FoTsc)]Cl_2_, **6**, and [Pd(FoTsc)_2_], **7**, were prepared (see Scheme 1) by some of us, as described in the literature [13, 17, 18].

All the compounds were firstly dissolved in DMSO (Koch-Light Laboratories Ltd, England) till the concentration of 1 M (stock solutions). Serial tenfold dilutions (100–0.000001 *μ*M) were made from them in cells growth medium DMEM (Gibco, USA) supplemented with 5% bovine serum (BS; BioWhittaker, Germany) and antibiotics (penicillin G, 100 units/mL, Balkanpharma, Bulgaria).

### 2.2. Cells and viruses

Continuous Madin-Darbey bovine kidney (MDBK) cells were used in the experiments. The cells were grown at 37°C in DMEM medium supplemented with 10% BS and antibiotics. During the experimentations, BS content was reduced to 5%. Antiviral experiments were done on the following four viruses: two wild (*wt*) strains Victoria (HSV-1) and BJA (HSV-2) and two ACV^R^ mutants with different TK gene mutations R-100 (TK^A^, HSV-1) and PU (TK^N^, HSV-2). Viruses were grown in MDBK cell monolayers. Cultures were harvested at full cytopathic effect (CPE), froze, thawed, and stored at −70°C.

### 2.3. Cytotoxicity and (HSV) assays

Confluent cell monolayers were washed and covered with media containing the compounds and cultured at 37°C for 48 hours. Cytopathic effect (CPE) was read by microscopy of unstained cell monolayers. Cell number was counted by the Trypan blue-dye exclusion method.

The cytotoxic concentration CC_50_ (concentration preventing the death of 50% of cells) and the maximal nontoxic concentration, MNC, were calculated from dose-response curves. The maximal concentration causing no cytotoxicity which does not alter the morphology of monolayers and the cell survival rate was recognized as MNC.

The antiviral activity of the complexes **1–7** against HSV replication was evaluated on the basis of their effects on the infectious HSV titer. MDBK cells were grown in 96-well plates and were infected with particular virus in serial tenfold dilutions. After 1 hour of virus attachment, infected cells were covered with medium and the tested compound in serial tenfold dilutions (starting from MNC) and cultured at 37°C for 48 hours (for *wt* strains) or 72 hours (for ACV^R^ mutants). Inhibitory concentrations required to inhibit virus yield by 50% (IC_50_) were calculated from dose-regression curves and were indicative for anti-HSV activity. In order to be able to compare the compounds on the basis of their selective inhibition of virus replication versus cytotoxicity, selective indexes (SI) were calculated as CC_50_ to IC_50_ ratio. The data were compared with that of ACV.

### 2.4. Direct PCR for determination of the effect on the expression of the immediate early (IE) reiterating region IV (ReIV)

Infected and mocked infected cells cultured in compound-free medium served as controls. PCR amplification 22 bp primers (Applied Biosystems, Calif, USA) were designed according to Maertzdorf et al. [21] to amplify 300 bp Us1 ReIV region of HSV-1 genome positions 132333-132634. The sequences (5′ → 3′) of the primer were ReIVUs1F-5′TCCGACGACAGAAACCCACC3′ and ReIVUs1R-5′GTCCCGGAGGACCACAGTGG3′. PCR was performed in a ready-to-go-PCR beads thermocycle (Amersham-Pharma Biotech, NJ, USA).

A 2 *μ*l sample of DNA suspension was added to the reaction mixtures and was overlaid with 25 *μ*l of mineral oil (CinnaGen Inc, Iran). PCR amplification was carried out as follows: an initial denaturation step of 94°C for 5 minutes followed by 35 cycles of alternating denaturation (94°C for 30 seconds), primer annealing (60°C for 60 seconds), and primer extension (72°C for 60 seconds). A final extension step of 5 minutes at 72°C was included. The PCRs were performed in 10 *μ*l volume. Briefly, 2 *μ*l of each sample were added to a separate tube containing 100 *μ*l of lysis buffer (Applied Biosystems) and stored at −20°C over night. After centrifugation at 12000 rpm for 5 minutes, the lysis buffer was removed, pellets were resuspended in nucleolysis buffer (300 *μ*l) phenol (Sigma Corporation of America, NY, USA), pH 7.8; 300 *μ*l chloroform: isoamyl alcohol = 24 : 1 (Sigma Corporation of America), and centrifuged at 12000 rpm for 5 minutes. DNA was extracted by resuspending the pellets in 10% SDS (Sigma Corporation of America) 10 mg/mL proteinase K (Pharma Biotech, USA), 10 mM Tris (Sigma Corporation of America ) and 0.1 mM EDTA (Sigma Corporation of America) at pH 7.4 and centrifuged at 12000 rpm for 5 minutes. A volume of 2 *μ*l of supernatants containing 50–100 ng of the resulting DNA suspension was used per PCR mixture. The reaction mixture contained 5 U/*μ*l cloned recombinant thermostable STS DNA taq polymerase (Applied Biosystems), corresponding primers at a concentration of 20 *μ*l/mL each, and 5 mM/*μ*l deoxynucleoside triphosphate (Pharma Biotech). Amplicons were electrophoresed on a 2% agarose gel and were visualized by ethidium bromide staining.

### 2.5. Apoptosis/necrosis in the noninfected cells and in cells infected with HSV

The staining methods of one-chain double-helices DNA have been used with 0.1% solution of acridine orange, and for mitochondria a solution of 0.1% of Janus green B has been used. An eukaryotic model of cells infected with HSV virus was used and the following modifications in the purpose of adapting the method to the corresponding system were adapted: (1) fixing of the cell with methanol not with formaldehyde; (2) after a standard procedure of staining in view of a further conservation of the preparations, treating with glycerol PBS = 1 : 1 was used. The experiments were carried out at the 8 hours of infection in the initial period of active virus morphogenesis. The following have been used as controls: (1) cells not infected and untreated with the investigated compounds; (2) cells not infected but treated with compounds; (3) cells infected with HSV and cultivated in a medium without an inhibitor.

The indicators for the lack of apoptosis/necroses were (1) the apple green fluorescence of cytoplasm and the nuclei according to the staining test with acridine orange; (2) a diffuse distribution of mitochondrial, glowing in green according to the staining test with Janus green B.

The indicators of apoptosis were a glowing of the nuclei in yellowish-red on a glowing of the cytoplasm in yellowish-green; a margination of chromatin; and an ejaculation of the nucleus content.

## 3. RESULTS AND DISCUSSION

CC_50_ and MNC values were calculated from dose-response curves and were presented in Table 1. All the compounds **1–7** exhibit lower cytotoxicity than ACV. MNC ranged from 1–100 *μ*M. Among them, higher cytotoxicity exhibits [Pt(FoTsc)_2_] and [Pd(FoTsc)Cl]. These two complexes are 50 times, [Pd(FoTsc)(H_2_FoTsc)]Cl_2_ and [Pd(FoTsc)_2_] are 500 times, and HFoTsc, [Pt(FoTsc)Cl] and [Pt(FoTsc)(H_2_FoTsc)]Cl_2_ are 5000 times less cytotoxic than ACV. The less cytotoxic compounds were HFoTsc (**1**) and its Pt(II) complexes **2** and **3**. The structure of **3** corresponds to [M(FoTsc)(H_2_FoTsc)]X_2_ [22–24]. Obviously, the protonated ligand in zwitterion form H_2_FoTsc^+^ decreases the cytotoxicity of Pt(II) but not of Pd(II), complex **6**.

The data presented on Table 1 show that the cytotoxicity of compounds **1–7** was predetermined by complex specificities.

The activity of compounds **1–7** was evaluated against *wt* HSV-1, strain Victoria, infection in cultured cells, and the data were compared to that of ACV. The complex **5** did not exhibit any effect on the infectious virus yield and it was excluded from further investigations. The rest of the compounds **1–4**, **6–7** were further evaluated against *wt* HSV-2 strain BJA and two ACV^R^ mutants with different TK gene mutations-R-100 (TK^A^) and PU (TK^N^), see Table 2.

The compounds **1–4** and **6-7** effectively inhibited the growth of *wt* and of ACV^R^, HSV-1 and HSV-2 strains and the effect were found to be predetermined by both complex and virus specificities. The most effective inhibitor of the *wt* HSV-1 growth was the ligand **1** while complex **4** was most sensitive to *wt* HSV-2. On the contrary, the growth of ACV^R^ viruses was effectively suppressed by the complexes **2** and **6**. The complexes of Pt(II) and Pd(II) and HFoTsc are arranged according to their efficacy against all four HSV strains in the following order:

- for *wt* HSV-1: **1** > **4** > **3** > **2** >** 7** ≫ **6**;
- for *wt* HSV-2: **4** > **6** > **1** = **3** ≫ **2** > **7**;
- for ACV^R^ mutants R-100 and PU: **2** = **6** ≫ **4** > **3** = **7**.

The selectivity of compounds **1–7** is shown in Table 2 and it was found to be predetermined by both complex and virus specificities. Complexes **1–7** are arranged according to their selectivity in the following order against all four strains:

- for *wt* HSV-1: **1** > **3** ≥ ACV > **4** > **2** ≥ ** 7 ** ≫ **6**;
- for *wt* HSV-2: **3** > **4** > ACV > **1** ≥ **6** ≫ **2** > **7**;
- for ACV^R^ strain R-100: **4** > **2** = **6** > **3** > ACV > **1** > **7**;
- for ACV^R^ strain PU: **2** > **3** > **6** = ACV > **1** > **7** = **4**.

The complex **3** was more sensitive to *wt* HSV strains, while the complex **2** was more sensitive to ACV^R^ mutants. The complex **7** was the less active and selective inhibitor of HSV replication and the complex **3** selectively inhibited the replication of both *wt* and ACV^R^ viruses.

The significant activity and selectivity of **3** are probably due to the negative influence on several viral targets. This is based on the fact that in solution, [M(FoTsc)(H_2_FoTsc)]X_2_ complexes dissociate to the metal complex [M(FoTsc)Cl] and the protonated ligand H_2_FoTscCl^+^ [22, 23], thus simultaneously suppressing virus-specific RR and the synthesis of DNA progeny.

Virus-specific proteins were identified on the 15 hours by Western blot analysis. Eleven virus-specific proteins were identified in viral control: VP5, VP22, VP23, *α*-TIF, TK, gB, gC, gE, gD, gH, and gG. In the compounds **1**, **3**, and **4**, VP23, TK, gG/gD, *α*-TIF, gH, and gE were not identified, see Table 3. These data suggest that compounds **1**, **3**, and **4** also suppress the morphogenesis, cell-to-cell spread, and transactivation of virus genomes.

**Figure 1:**
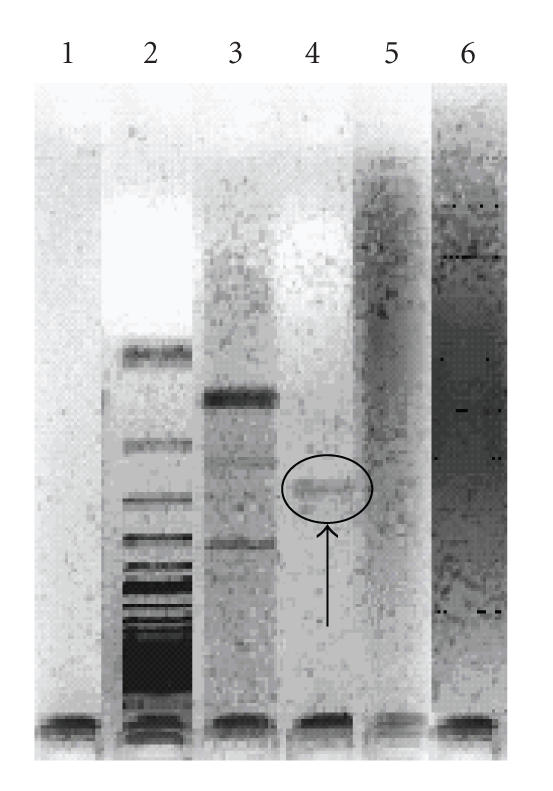
Amplification of ReIV containing region within *wt* HSV-1 genome. (1) negative control (lane 1); (2) markers (lane 2); (3) cell control (mock-infected cells cultured in compound-free medium) (lane 3); (4) positive control (HSV-1-infected cells cultured in compound-free medium) (lane 4); (5) [Pt(FoTsc)(H_2_FoTsc)]Cl_2_, **3** (lane 5), and (6) HFoTsc, **1** (lane 6).

In view of the fact that HFoTsc and **3** are not only effective and selective HSV inhibitors, but they also suppress the expression of the essential structure proteins from the *β*(E) and *γ*(L) kinetic groups, whose synthesis is impossible without *α*, IE proteins, the effect of **3** over the expression of the immediately earliest *α*, IE genes by means of a direct PCR was studied. A direct multiplication was used with PCR by a primer, determining region 300 bp, corresponding to ReIV region of Us1. The results of the gel electrophoresis of DNA extracted from viral infected control cells and treated for 2 hours after infection with the HSV-1 with MNC of [Pt(FoTsc)(H_2_FoTsc)]Cl_2_, **3** and HFoTsc and **1** are shown in Figure 1. Just as expected, it was observed that the DNA of control MDBK cells appears as a band corresponding to genomic DNA (Figure 1, lane 3). Incubation of the HSV-1 infected cells with the MNC of [Pt(FoTsc)(H_2_FoTsc)]Cl_2_, **3** or the HFoTsc, **1** ligand resulted in a “DNA smears,” which shows the nonspecific fragmentation of DNA (Figure 1, lanes 5 and 6, resp.). The results received by direct PCR show that [Pt(FoTsc)(H_2_FoTsc)]Cl_2_, **3** (lane 5), and the ligand HFoTsc **1** (lane 6) suppress the expression of the *α*, IE virus genes. Altogether, these data suggest that in MDBK infected cells, the nonspecific destruction of viral DNA is obviously caused by Pt(II) ions [25, 26] and may be due to a specific induction of apoptosis.

The effect of **3** on programmed cell death was evaluated morphologically in order to study if the observed DNA fragmentation is cell- and/or virus-specific. Using acridine orange and Janus B green staining morphological changes leading to irreparable margination of chromatin, an ejaculation of the nucleus content and other indicators for apoptosis were not found in *wt* HSV-1 nor in mock-infected cells on the 8 hours after the action of [Pt(FoTsc)(H_2_FoTsc)]Cl_2_, **3**. It was observed that complex **3** specifically affects HSV replication simultaneously suppressing virus-specific RR and DNA polymerase and the expression of virus genome immediately after entering host cell nucleus. This also explains the nonspecific virus response to [Pt(FoTsc)(H_2_FoTsc)]Cl_2_.

The experimental data show that [Pt(FoTsc)(H_2_FoTsc)] · Cl_2_ complex is a promising anti-HSV agent which could be useful in the treatment of HSV infections, especially when the causative agent is resistant to ACV mutants. The platinum complex [Pt(FoTsc)(H_2_FoTsc)]Cl_2_ decreases the cytotoxicity of Pt(II) ion and directs its activity to viral and not to host cell DNA.

## Figures and Tables

**Table 1:** MNC, CC_50_, and IC_50_ (in *μ*M) of HFoTsc and its Pt(II) and Pd(II) metal complexes; NA: not active and ND: not done.

Number	Compound	CC_50_	MNC	Victoria	BJA	IC_50_ R-100	PU

**1**	HFoTsc	830	100	0.001	0.1	1	1
**2**	[Pt(FoTsc)Cl]	548	100	0.1	5	0.1	0.1
**3**	[Pt(FoTsc)(H_2_FoTsc)]Cl_2_	5820	100	0.01	0.1	1	1
**4**	[Pt(FoTsc)_2_]	40	1	0.006	0.001	0.5	0.5
**5**	[Pd(FoTsc)Cl]	69	1	NA	ND	ND	ND
**6**	[Pd(FoTsc)(H_2_FoTsc)]Cl_2_	63	10	10	0.01	0.1	0.1
**7**	[Pd(FoTsc)_2_]	59	10	0.06	1	1	1
**8**	ACV	25	0.02	0.0002	0.002	0.02	0.02

**Table 2:** Selectivity of HFoTsc and its Pt(II) and Pd(II) complexes against HSV infection in cultured cells.

Number	Compound	Selective index (SI)			
		Victoria	BJA	R-100	PU

**1**	HFoTsc	830 000	7000	900	700
**2**	[Pt(FoTsc)Cl]	5480	110	6400	6200
**3**	[Pt(FoTsc)(H_2_FoTsc)]Cl_2_	582000	52000	5300	5200
**4**	[Pt(FoTsc)_2_]	6667	20 000	7200	40
**6**	[Pd(FoTsc)(H_2_FoTsc)]Cl_2_	6.3	6300	6300	1000
**7**	[Pd(FoTsc)_2_]	983	59	590	58
**8**	ACV	125000	10 000	1000	1000

**Table 3:** Data of **1**, **3**, and **4** for virus-specific proteins from Western blot analysis.

Protein	Kinetic group	MM, kd	Gene	**1**	**3**	**4**	HSV-1 control

VP26	*γ*2	12	U_L_35	+	+	+	+
VP24	*γ*	25	U 49	−	+	+	−
VP22	—	29	U_L_26	−	−	+	+
VP22/22a	*γ*1	39–50	U_L_48	−	+	+	+
*α*-TIF	*γ*1	53	U_s_4/	+	−	−	+
gG/gD	*γ*1	59	U_s_6	−	+	−	+
VP11	*γ*1	71	U_L_46	−	+	−	+
gE	*γ*1	90	U_s_8	−	+	+	+
gH	*γ*2	110–115	U_L_22	+	+	+	−
gC	*γ*2	130	U_L_44	+	+	—	+
VP5	*γ*1	155	U_L_19	+	+	+	+
Pre-	*β*2/*γ*1	178–195	U_L_27	+	−	+	−
gB	*β*2/*γ*1	240	U_L_27	+	+	+	+
